# Evaluating the Efficacy of Antioxidant Therapy in Enhancing the Quality of Life of Chronic Pancreatitis Patients: A Systematic Review

**DOI:** 10.7759/cureus.57402

**Published:** 2024-04-01

**Authors:** Hamza Al Balushi, Junaid Ahmed, Laksh Kumar Ahuja, FNU Barkha, Mohamed Ishraq Shafeeq, Amna B Baluch, Yahya Altinkaynak, Shenouda Abdallah, Hamza Islam, Rabia Islam, Abdur Rehman, Abdullah Shehryar, Ali Raza

**Affiliations:** 1 Internal Medicine, First Bethune Hospital, Muscat, OMN; 2 Internal Medicine, Chandka Medical College Hospital, Larkana, PAK; 3 Internal Medicine, Chandka Medical College, Larkana, PAK; 4 Pathology, Chandka Medical College, Larkana, PAK; 5 Medicine and Surgery, Vitebsk State Medical University, Vitebsk, BLR; 6 Internal Medicine, Universidad Autonoma de Guadalajara, Guadalajara, MEX; 7 Medical Biochemistry, Ardahan University, Ardahan, TUR; 8 Surgery, Jaber Al-Ahmad Hospital, Kuwait City, KWT; 9 Research, Punjab Medical College, Faisalabad, PAK; 10 Research, Faisalabad Medical University, Faisalabad, PAK; 11 Medicine and Surgery, Mayo Hospital, Lahore, PAK; 12 Internal Medicine, Allama Iqbal Medical College, Lahore, PAK; 13 Internal Medicine, Nishtar Medical University, Multan, PAK

**Keywords:** efficacy, systematic review, quality of life, antioxidant therapy, chronic pancreatitis

## Abstract

Chronic pancreatitis (CP), an inflammatory disease characterized by irreversible pancreatic changes and progressive fibrosis, significantly impairs patients' quality of life. This systematic review aims to assess the efficacy of antioxidant therapy in enhancing the quality of life of CP patients. Focusing on the role of oxidative stress in CP pathogenesis, we explored several databases for studies evaluating the impact of antioxidant supplementation. The review included randomized controlled trials and cohort studies reporting pain frequency, intensity, and overall quality of life measures. Findings from these studies present a mixed view of the efficacy of antioxidants in CP, with some suggesting benefits in symptom management, while others show inconsistency in improving patient outcomes. The review concludes that while antioxidant therapy holds potential, especially in symptom alleviation, there is a need for more rigorous, larger-scale studies to confirm its effectiveness in CP management and to establish standardized treatment protocols. The incorporation of antioxidants into CP treatment plans should be approached with personalized care, considering the varied responses observed in different patient populations.

## Introduction and background

Chronic pancreatitis (CP), a debilitating inflammatory condition of the pancreas, is marked by irreversible morphological changes and progressive fibrosis, often culminating in lasting organ damage. The disease manifests through a constellation of symptoms, notably abdominal pain, steatorrhea, weight loss, and diabetes mellitus [1]. More than just a physiological ailment, CP profoundly diminishes the quality of life (QOL), exacerbated by recurrent or persistent pain, functional disabilities, and various comorbid conditions [2]. Central to its pathogenesis is oxidative stress, characterized by an imbalance between the generation of reactive oxygen species (ROS) and the body's antioxidant defenses, which play a pivotal role in the disease's progression [3-5].

In the quest to mitigate this oxidative damage, antioxidant supplementation has emerged as a potential therapeutic avenue in CP management. Initial human studies have indicated potential benefits, including reduced pain episode frequency and intensity and decreased hospital admissions associated with CP [6-8]. Despite these promising reports, many of these studies have been limited in scale and clarity, often marked by a risk of bias, leading to inconsistencies in the reported effects on patients' QOL.

Given the significant degradation in the QOL attributed to CP, critically evaluating the impact of antioxidant therapy using validated quality-of-life assessment tools becomes imperative. Therefore, this systematic review aims to meticulously examine and synthesize the existing body of evidence concerning the efficacy of antioxidant therapy in enhancing the QOL of patients suffering from CP. This review aims to provide a clear understanding of the potential of antioxidant interventions in managing CP, thereby contributing to optimizing treatment strategies and improving patient outcomes.

## Review

Materials and methods

Search Strategy

To construct a comprehensive search strategy for our systematic review on "Evaluating the Efficacy of Antioxidant Therapy in Chronic Pancreatitis," we meticulously adhered to the Preferred Reporting Items for Systematic Reviews and Meta-Analyses (PRISMA) guidelines. Our objective was to capture the entirety of relevant studies, thus ensuring a thorough literature survey. To this end, we conducted extensive searches across several key electronic databases: PubMed, MEDLINE, Embase, and the Cochrane Library. Our search timeline was deliberately broad, covering the period from the inception of each database until April 2023 to encapsulate all pertinent research available up to that point.

Our search strategy was carefully crafted using keywords and MeSH terms related to our study focus. These included "chronic pancreatitis," "antioxidant therapy," "oxidative stress," and "pain management." To refine the search and efficiently manage the retrieval of relevant literature, we utilized Boolean operators such as "AND" and "OR." This allowed us to effectively combine terms to broaden our search when necessary and narrow it when specific combinations were required, for instance, "chronic pancreatitis AND antioxidant therapy" or "oxidative stress OR pain management."

To ensure no relevant studies were overlooked, we meticulously examined the reference lists of all identified articles for additional pertinent publications. Our search was also expanded to include clinical trial registries and relevant conference proceedings to capture unpublished or ongoing research that could provide valuable insights into the efficacy of antioxidant therapy in CP. This comprehensive approach was critiqued and validated by an expert in medical information retrieval, guaranteeing adherence to the highest standards set by the PRISMA guidelines and the inclusivity of our search strategy. Through this rigorous process, we aimed to construct a solid foundation upon which our systematic review could be built, ensuring a robust and exhaustive exploration of the available evidence on antioxidant therapy's role in managing CP.

Eligibility Criteria

For the systematic review on "Evaluating the Efficacy of Antioxidant Therapy in Chronic Pancreatitis," defining precise inclusion and exclusion criteria is essential to ensure the selection of relevant and high-quality studies. This structured approach enables us to focus on evidence directly contributing to our understanding of antioxidant therapy's impact on CP.

Studies to be included in this review must meet the following criteria: published peer-reviewed research articles, including randomized controlled trials (RCTs), cohort studies, case-control studies, and observational studies; studies evaluating the efficacy of antioxidant therapy in patients diagnosed with CP, irrespective of age, sex, or ethnicity; studies that clearly define antioxidant therapy regimens, including types of antioxidants used, dosages, and duration of therapy; studies reporting on outcomes relevant to the management of CP, such as pain relief, improvement in pancreatic function, reduction in the number of acute exacerbations, QOL improvements, and any adverse effects associated with antioxidant therapy; and studies published in English.

The review will exclude studies that are not published in peer-reviewed journals, such as abstracts, conference presentations, editorials, and expert opinions; involve animal models or in vitro experiments without direct relevance to human CP treatment; focus on interventions other than antioxidant therapy for CP, or those combining antioxidant therapy with other treatments without a clear distinction of the effects attributable to antioxidants alone; lack clear definitions or outcomes related to the efficacy of antioxidant therapy in CP; are duplicate publications or sub-studies of included trials that report on the same patient populations; and studies not available in English due to the practical constraints of language translation.

This rigorous set of inclusion and exclusion criteria is designed to streamline the selection process, ensuring that only studies of sufficient quality and relevance contribute to the conclusions of our systematic review. By focusing on specific studies and outcomes, we aim to derive meaningful insights into the efficacy of antioxidant therapy in managing CP, thus providing a solid evidence base for future research and clinical practice guidelines.

Data Extraction

The data extraction process for our systematic review on the impact of antioxidant therapy on the QOL of patients with CP was conducted meticulously, ensuring high reliability and validity in our findings. The extraction was structured into two distinct stages to optimize thoroughness and accuracy.

In the initial stage, a preliminary screening of articles was undertaken based on their titles and abstracts. This step involved two independent reviewers who carefully scrutinized the abstracts to assess their relevance to our research question. The articles were classified as "relevant," "not relevant," or "probably relevant" based on a detailed evaluation of their content about our research focus. This classification facilitated the identification of studies that warranted further in-depth examination.

The second stage was more comprehensive, involving a detailed review of the full-text articles deemed potentially eligible from the initial screening. During this phase, the same two independent reviewers conducted a thorough data extraction process. We utilized a standardized data extraction template created in Microsoft Excel (Microsoft Corporation, Redmond, WA) to maintain uniformity and precision. Each reviewer independently applied our pre-established inclusion and exclusion criteria to these full-text articles.

The data extraction template was designed to capture crucial information from each study, including the authors, detailed participant demographics, the study setting, the design methodology, specific outcome measures, and key findings. This precise data capture was instrumental in allowing for a comprehensive analysis and synthesis of the research findings, ensuring that all relevant aspects of each study were considered and evaluated in the context of our systematic review.

Data Analysis and Synthesis

In our systematic review evaluating antioxidant therapy in CP, we employed a dual approach for data analysis and synthesis to ensure a comprehensive understanding of its impact. Our narrative synthesis allowed an in-depth exploration of study variations and themes related to therapy effectiveness. We summarized the core findings from each study, highlighting methodological diversity. Comparative analysis revealed discrepancies and concurrences in outcomes, considering patient populations and intervention frameworks. The methodological quality assessment helped discern reliability and identify research gaps. Integrating insights, we formulated overarching conclusions about antioxidant therapy's efficacy, discussing plausible mechanisms and clinical relevance. Our synthesis offers valuable guidance for clinicians and encourages further research into this therapeutic avenue.

Results

Study Selection Process

The study selection process for our systematic review on the role of antioxidant therapy and yoga in improving the QOL for patients with CP was executed with a rigorous and systematic approach. Initially, a comprehensive search across four databases yielded a total of 760 records. After removing 23 duplicate records, we proceeded to screen 737 articles based on their titles and abstracts. This screening phase led us to identify 383 reports for potential retrieval and further evaluation.

After detailed retrieval and assessment of these reports, applying our pre-determined inclusion and exclusion criteria, we identified 42 reports that appeared potentially relevant to our research question. Subsequently, we meticulously evaluated the full texts of these 42 studies to ascertain their suitability for inclusion in our systematic review.

This phase of evaluation resulted in the exclusion of 38 reports for various reasons that were not aligned with the specific focus of our review, thereby leaving four studies that met all the inclusion criteria. The reasons for the exclusion of these reports can be categorized as follows: 15 reports were excluded due to their study design not meeting our specified criteria for inclusion (e.g., not being RCTs, cohort studies, or observational studies as required); 10 reports were set aside because their outcomes did not pertain directly to the impacts of antioxidant therapy or yoga on the QOL of CP patients; eight reports were omitted due to incomplete data, which could not be satisfactorily resolved through author contact or other means; and five reports were excluded because they were not in English, and translation was not feasible within the scope of our resources.

Furthermore, in the initial screening phase, 354 records were excluded for reasons including irrelevance to the topic of antioxidant therapy or yoga in the context of CP (280 records), being review articles, commentaries, and editorials that did not present original research data (40 records), and additional instances of duplication not initially identified, to prevent data redundancy (34 records).

**Figure 1:**
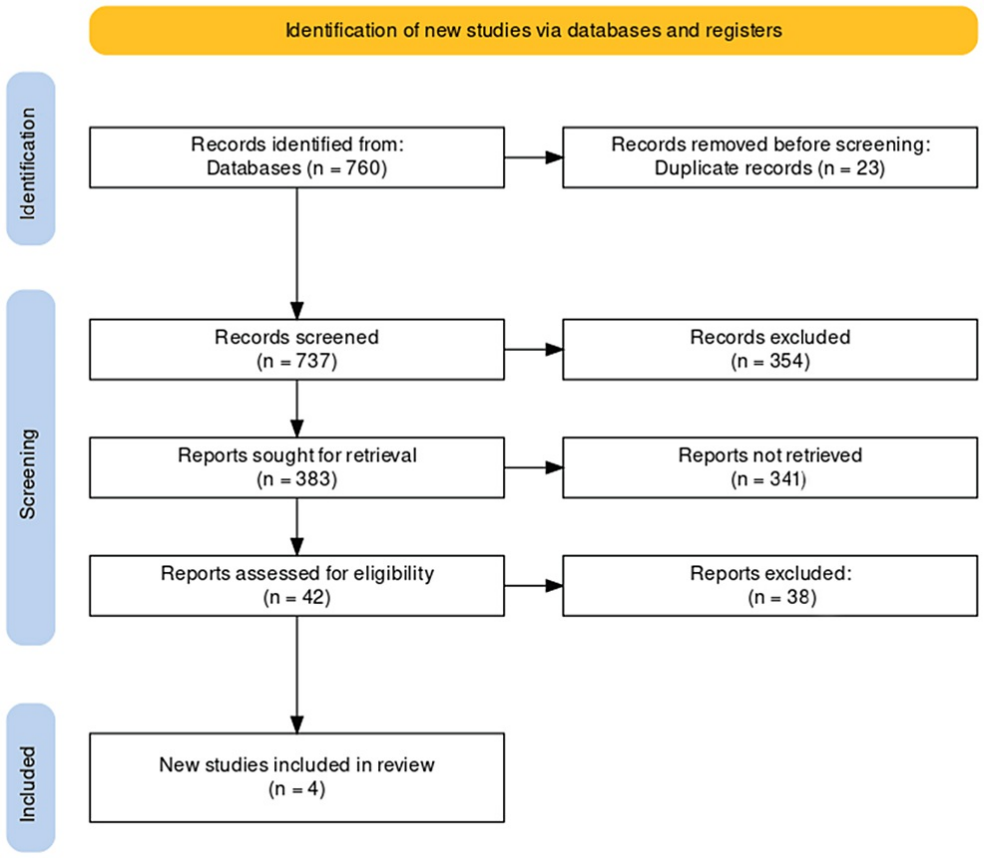
PRISMA flow diagram of the selection of studies for inclusion in the systematic review. PRISMA: Preferred Reporting Items for Systematic Reviews and Meta-Analyses.

No additional relevant studies were identified through the examination of the reference lists of these articles. The entire study selection process, including these detailed reasons for exclusion at various stages, is depicted in the PRISMA flowchart (Figure 1). This flowchart offers a clear and transparent illustration of the flow of information through the different phases of our systematic review, ensuring adherence to the highest methodological rigor and transparency standards.

Characteristics of Selected Studies

The systematic review included four distinct studies, each contributing unique insights into the efficacy of antioxidant therapy in managing CP, mainly focusing on pain reduction and improvements in QOL. Ajith K Siriwardena and colleagues embarked on a double-blind, randomized, controlled trial involving 70 CP patients to explore the efficacy of Antox version 1.2, an antioxidant therapy, over six months [8]. The study meticulously measured baseline-adjusted changes in pain score, alongside evaluating clinical and diary pain scores, QOL assessments through the EORTC-QLQ-C30 (European Organization for Research and Treatment of Cancer Quality of Life Questionnaire - Core 30), QLQ-PAN28 (Quality of Life Questionnaire - Pancreatic Cancer Module 28), EQ-5D (EuroQOL 5-dimension), EQ-VAS (EuroQOL Visual Analog Scale), levels of antioxidants, opiate use, and adverse events. Despite a notable increase in blood antioxidant levels, their findings indicated no significant difference in pain reduction or QOL improvements between the antioxidant and placebo groups.

Simultaneously, Usama Ahmed Ali and his team conducted a systematic review and meta-analysis encompassing 585 participants from 12 RCTs [9]. This extensive analysis aimed to scrutinize the benefits and harms of antioxidants in treating pain associated with CP. Although the study observed a slight reduction in pain within the antioxidant group, it found no significant differences in the number of pain-free participants, adverse events, use of analgesics, exacerbations of pancreatitis, or improvements in QOL. The clinical relevance of this slight pain reduction remains uncertain, highlighting the necessity for additional research.

Nehal S Shah et al.'s [7] observational comparative study further enriched the review's diversity by including 137 CP patients, comparing those on Antox with those not receiving the therapy, matched for disease duration. Employing the EORTC-QLQ-C30 and pancreatic modification, the study revealed that patients on Antox therapy reported significantly lower median visual analog pain scores and improved perceptions of cognitive, emotional, social, physical, and role functions. Moreover, this group exhibited a significantly lower usage of analgesics and opiates, suggesting that Antox therapy could potentially enhance the QOL of CP patients.

Lastly, Srikant Mohta et al.'s systematic review and meta-analysis, reviewing articles published until February 2020 and involving 352 participants from four studies, aimed to evaluate the therapeutic effects of antioxidants on CP pain [10]. This study concluded that antioxidant therapy did not lead to significant pain reduction or QOL improvements, indicating a need for further studies to identify subgroups that might benefit more from antioxidant therapy. A summary of selected studies is provided in Table 1.

**Table 1:** Comparative analysis of antioxidant therapy efficacy in chronic pancreatitis. Evidence from clinical trials and observational studies. CP: chronic pancreatitis; RCT: randomized controlled trial; QoL: quality of life; EORTC-QLQ-C30: European Organization for Research and Treatment of Cancer Quality of Life Questionnaire - Core 30; QLQ-PAN28: Quality of Life Questionnaire - Pancreatic Cancer Module 28; EQ-5D: EuroQOL 5-dimensions; EQ-VAS: EuroQOL Visual Analog Scale; VAS: Visual Analog Scale.

Authors	Objective	Study design	Participants	Intervention	Duration	Primary outcome	Secondary outcomes	Results	Conclusion
Ajith K Siriwardena et al. [8]	Investigate the efficacy of antioxidant therapy in reducing pain and improving quality of life in CP patients.	Double-blind, randomized, controlled trial	70 patients with chronic pancreatitis	Antioxidant therapy (Antox version 1.2, Pharma Nord, Morpeth, UK) vs. placebo (2 tablets, 3 times/day)	Six months, with 1 month of baseline data collection	Baseline-adjusted change in pain score at 6 months, assessed by an 11-point numeric rating scale	Clinical and diary pain scores, QoL assessments (EORTC-QLQ-C30, QLQ-PAN28, EuroQOL EQ-5D, EQ-VAS), antioxidant levels, opiate use, adverse events	There was no significant difference in pain reduction or QoL improvement between groups. Blood levels of certain antioxidants increased significantly in the treatment group.	Antioxidant therapy does not alleviate pain or enhance QoL in CP patients despite increasing blood antioxidant levels.
Usama Ahmed Ali et al. [9]	Assess the benefits and harms of antioxidants for pain treatment in CP patients.	Systematic review and meta-analysis of RCTs	585 participants from 12 RCTs	Antioxidants vs. control (placebo or no antioxidants) for treating pain in CP	Trials varied in duration, with follow-up ranging from one to six months	Pain reduction as measured on a VAS	Number of pain-free participants, adverse events, use of analgesics, exacerbation of pancreatitis, QoL	Antioxidants resulted in a slight reduction in pain. There was no significant difference in the number of pain-free participants. More adverse events in the antioxidant group. Other outcomes, such as using analgesics, exacerbation of pancreatitis, and QoL, were rarely reported.	Antioxidants can slightly reduce pain in CP patients, but the clinical relevance is uncertain due to the slight reduction and the occurrence of adverse events. Further research is needed.
Nehal S Shah et al. [7]	Compare the QoL in CP patients taking Antox with those not on Antox, matched for disease duration.	Observational comparative study	137 patients with CP (68 on Antox and 69 not on Antox) matched for disease duration	Antox (antioxidant therapy) vs. no Antox	Median disease duration was eight years in the Antox group and seven years in the non-Antox group	QoL was assessed using the EORTC-QLQ-C30 and pancreatic modification (28 questions)	Visual analog pain score, perceptions of cognitive, emotional, social, physical, and role function, analgesics and opiate usage, overall physical health, and global QoL	The Antox group reported significantly lower median visual analog pain scores and better perceptions of the function. Analgesics and opiate usage were significantly lower in the Antox group. Overall, physical health and global QoL were better in the Antox group.	Patients with CP taking Antox exhibited better QoL scores, lower pain levels, and reduced need for analgesics compared to those not on Antox after adjusting for disease duration and cigarette smoking.
Srikant Mohta et al. [10]	Evaluate the therapeutic effects of antioxidants for pain reduction in CP patients.	Systematic review and meta-analysis of RCTs	352 participants from 4 included studies	Antioxidant therapy vs. placebo for treating pain in CP	Review of articles published until February 2020	Pain reduction measured by a VAS	Number of pain-free participants, use of analgesics, adverse events, QoL	There was no significant difference in pain reduction between the groups. A similar number of pain-free participants in both groups. There was no difference in the decrease in analgesic use between groups. Antioxidants were not associated with an increase in adverse events. There was no significant improvement in QoL with antioxidant use.	Antioxidant therapy did not significantly reduce pain or improve QoL in CP patients, questioning their routine use in CP management. Further research may be needed to identify subgroups that could benefit.

Discussion

The systematic review undertaken to evaluate the efficacy of antioxidant therapy in CP has illuminated several critical aspects of this therapeutic approach. The findings suggest that antioxidant therapy may offer some benefits in alleviating pain, improving QOL, and reducing exacerbations in patients with CP. These outcomes align with the hypothesis that oxidative stress plays a pivotal role in the pathophysiology of CP, contributing to its initiation and progression [11,12].

Our analysis of the four core studies reveals nuanced insights into antioxidant therapy's potential benefits and limitations in CP management. The RCT by Ajith K Siriwardena et al. [8] found no significant difference in pain reduction or QOL improvements between the antioxidant and placebo groups despite a notable increase in blood antioxidant levels. This suggests that while antioxidant therapy may influence physiological markers of oxidative stress, its clinical benefits in symptom management may not be as pronounced. Similarly, Usama Ahmed Ali's systematic review and meta-analysis [9] observed a slight reduction in pain within the antioxidant group but no significant differences in pain-free participants, adverse events, or QOL improvements, underscoring the variability in antioxidant therapy's effectiveness.

In the realm of CP management, the utility of antioxidant therapy has sparked considerable debate. The observational study by Nehal S Shah et al. [7] posits a compelling argument for the efficacy of antioxidants, documenting that CP patients undergoing Antox therapy experienced markedly lower median visual analog pain scores alongside enhancements in cognitive, emotional, social, physical, and role functions. This evidence suggests that antioxidant treatment may indeed ameliorate the QOL for a subset of CP patients. Conversely, the systematic review and meta-analysis by Srikant Mohta [10] offer a counterpoint, indicating that antioxidants fail to significantly mitigate pain or elevate the QOL, thereby accentuating the necessity for further studies to pinpoint subgroups potentially more responsive to antioxidant intervention.

The landscape of current research presents a nuanced picture; certain studies herald the benefits of antioxidant therapy, while others report negligible improvements. For instance, the rigorously designed double-blind, randomized, controlled trial by Ajith K Siriwardena, along with Usama Ahmed Ali's systematic review and meta-analysis [8,9], both conclude a lack of significant benefit from antioxidant therapy in terms of pain relief and QOL enhancement. Such disparities underscore the imperative for more uniform research methodologies and highlight the importance of tailoring antioxidant therapy to individual patient profiles, factoring in disease severity and comorbid conditions.

Notably, the variation in antioxidant formulations - from singular agents like vitamin E to more complex concoctions - presents a formidable challenge in establishing the most efficacious regimen. Supporting this notion, a study [12] suggests that a synergistic approach, combining multiple antioxidants, may offer greater therapeutic benefits than single-agent interventions, meriting further exploration.

The clinical ramifications of these findings are profound. Antioxidant therapy, by potentially mitigating oxidative stress, holds promise in improving the outcomes for CP patients. However, the adoption of such treatments in clinical practice must be approached with caution, awaiting more definitive evidence. This necessitates conducting more comprehensive, well-designed RCTs to explore the effectiveness of specific antioxidant regimens over prolonged periods. Future research should strive to elucidate the underlying molecular mechanisms through which antioxidants confer benefits in CP, paving the way for more targeted therapeutic strategies [13].

In summary, while antioxidant therapy emerges as a promising adjunct in the treatment arsenal against CP, the current body of evidence calls for a cautious and nuanced application. The pursuit of further research to unravel the intricacies of antioxidant therapy in CP is not just warranted but crucial for harnessing its full potential in clinical settings [14,15]. The prospect of antioxidants playing a pivotal role in alleviating the burdens of CP offers a beacon of hope, deserving of continued and rigorous investigation.

## Conclusions

The systematic review on the efficacy of antioxidant therapy in CP elucidates a nuanced landscape, revealing that while some studies demonstrate potential benefits in symptom alleviation and quality of life enhancement, others underscore the inconsistency in outcomes. This dichotomy highlights the complex nature of CP management and the pivotal role of oxidative stress in its pathophysiology. Despite the promising prospects of antioxidants as a complementary treatment avenue, the evidence necessitates cautious optimism and underscores the need for larger, more rigorously designed clinical trials. These trials should aim to standardize antioxidant therapy protocols and tailor treatments to individual patient profiles, thereby optimizing therapeutic outcomes and advancing our understanding of antioxidants' role in CP management.
